# Embolization of renal artery pseudoaneurysm following laparoscopic partial nephrectomy for central renal tumor: A report of two cases

**DOI:** 10.3892/ol.2014.2045

**Published:** 2014-04-08

**Authors:** HAO PAN, DAN XIA, SHUO WANG, ZHAN WANG, BAISHU ZHONG, XIANYONG ZHOU, ZHIYI PENG

**Affiliations:** 1 Department of Urology, The First Affiliated Hospital, Zhejiang University School of Medicine, Hangzhou, Zhejiang 310003, P.R. China; 2 Department of Radiology, The First Affiliated Hospital, Zhejiang University School of Medicine, Hangzhou, Zhejiang 310003, P.R. China

**Keywords:** laparoscopic partial nephrectomy, central renal tumor, renal artery pseudoaneurysm

## Abstract

Laparoscopic partial nephrectomy has recently emerged as a minimally invasive treatment for small- to moderate-sized renal tumors. Renal artery pseudoaneurysms (RAPs) have been well-reported in patients with renal trauma or who have undergone percutaneous urological procedures, including biopsy, nephrostomy and percutaneous nephroureterolithotomy. However, RAP following laparoscopic partial nephrectomy for central renal tumor is a rare but serious, potentially life-threatening complication. In total, two patients underwent laparoscopic partial nephrectomy at The First Affiliated Hospital of Zhejiang University School of Medicine (Hangzhou, China) for central renal tumors that had developed gross hematuria several weeks following the surgical procedures. The formation of RAPs was confirmed by contrast-enhanced computed tomography scans. Superselective embolizations of the renal artery branches were successfully performed to treat these two patients. In the current report, the etiology, diagnosis and management of RAPs are discussed.

## Introduction

Laparoscopic nephron-sparing surgery has been increasingly performed by urologists for patients with renal tumors, and its complications have received increasing attention (1). As a rare and potentially fatal complication, renal artery pseudoaneurysm (RAP) is often reported in patients undergoing percutaneous nephroscopy, renal trauma, renal transplantation and kidney biopsy. Recently, two patients underwent laparoscopic partial nephrectomy for central renal tumors at The First Affiliated Hospital of Zhejiang University School of Medicine (Hangzhou, China) who had developed RAPs following surgery. The aim of the present study was to introduce a novel therapeutic method, superselective embolization of the renal artery branches, which was used to cure these two patients. Written informed consent was obtained from the patients.

## Case report

### Laparoscopic surgical procedures

The two patients underwent four-port extraperitoneal laparoscopic procedures. Preoperative computed tomography (CT) angiography indicated only one main trunk of the renal artery of the affected kidney in each patient. Initially, a noninvasive vascular clip was used to block the main trunk of the renal artery. The edge of the tumor was identified and scissors were used to resect the tumor with a 5-mm margin. A 3-0 coated Vicryl^®^ (polyglactin 910) suture (Johnson & Johnson, New Brunswick, NJ, USA) was used to close the vascular section with the hemorrhage and collecting system. Then, 2-0 coated Vicryl (polyglactin 910) suture (Johnson & Johnson) and an absorbable clip were used to close the renal parenchyma wound. The noninvasive vascular clip was released and inactive bleeding was confirmed. The renal fossa drainage tubes of cases one and two were withdrawn seven and five days following surgery, respectively.

### Case one

**Figure 1:**
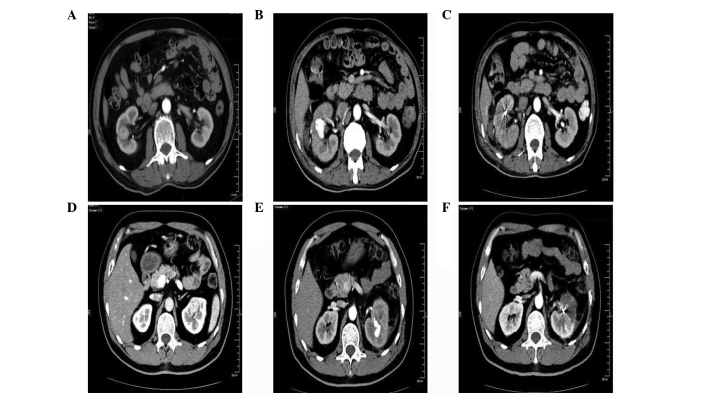
Different situations of the lesions of the two cases through physical examination. Case one showed: (A) A 2.×2.5-cm space-occupying lesion in the center of the lower pole of the right kidney parenchyma; (B) a 6-cm hematoma on the edge of the right kidney and a 2.2-cm cystic shadow bound to the surgical area of right kidney with enhancement; and (C) superselective embolizations of the renal artery branches. Case two showed: (D) A 2.5×3.0-cm space-occupying lesion in the center of the upper pole of the left kidney parenchyma; (E) a ~3-cm cystic shadow bound to the center of the upper pole of the left kidney with enhancement; and (F) superselective embolizations of the renal artery branches.

A 68-year-old male patient presented to The First Affiliated Hospital of Zhejiang University School of Medicine with a previous history of hypertension and diabetes. A 2.5×2.5-cm space-occupying lesion in the center of the lower pole of the right kidney parenchyma was identified during the physical examination (Fig. 1A). The results of the preoperative CT angiography indicated that the bilateral renal arteries had only one main trunk and no abnormal feeding arteries of the lesion. The results of the preoperative laboratory tests (liver function, kidney function, routine blood test and clotting time) were all within normal ranges. Laparoscopic surgery was performed as previously described. The warm ischemia time of the right kidney was 28 min and postoperative recovery of the patient was good. The pathological examination was performed to confirm renal cell carcinoma (clear cell type) and the negative margins.

On day 23 following surgery, the patient complained of gross total hematuria. The enhanced CT scan found a 6-cm hematoma on the edge of the right kidney and a 2.2-cm cystic shadow bound to the surgical area of the right kidney with enhancement. These results suggested the formation of a RAP (Fig. 1B). On day 25 following surgery, the selective embolization of the right renal artery branches failed. Thus, on day 29, the embolization was performed again and was successful. The hematuria was controlled and the results of the CT scan performed two weeks following the embolization indicated that the RAP had contracted (Fig. 1C).

### Case two

A 47-year-old male patient presented to The First Affiliated Hospital of Zhejiang University School of Medicine with a previous history of diabetes. A 2.5×3-cm space-occupying lesion in the center of the upper pole of the left kidney parenchyma was identified during a physical examination (Fig. 1D). The results of the preoperative CT angiography indicated that the bilateral renal arteries had only one main trunk and no abnormal feeding arteries of the lesion. The results of the preoperative laboratory tests (liver function, kidney function, routine blood test and clotting time) were all within normal ranges. Laparoscopic surgery was performed as previously described. The warm ischemia time of the left kidney was 31 min and postoperative recovery of the patient was good. The pathological examination was performed to confirm the angiomyolipoma.

On day 31 following surgery, the patient complained of gross total hematuria and left lumbago, and received symptomatic hemostasis at Xinchang Renmin Hospital (Xinchang, China), but the symptoms were not controlled. On day 36 following surgery, the patient was admitted to The First Affiliated Hospital of Zhejiang University School of Medicine. A contrast-enhanced CT scan revealed a ~3-cm cystic shadow bound to the center of the upper pole of the left kidney, with enhancement similar to that of the surrounding renal arteries, which suggested the formation of a RAP (Fig. 1E). On day 37 following surgery, the patient underwent selective embolization of the left renal artery branches. This procedure was successful and the patient’s hematuria was controlled. The results of the CT scan performed one week following the embolization indicated that the RAP had contracted (Fig. 1F).

## Discussion

In 1993, Winfield *et al* first reported laparoscopic partial nephrectomy for benign kidney disease (2). To date, this surgery has been increasingly used to treat patients with malignant renal tumors.

Rupture and hemorrhage of the renal artery or renal artery branch may lead to a hematoma, which may be wrapped by the surrounding tissue and form a cystic cavity that connects with the artery, a condition termed as RAP (3). The cyst does not have a normal arterial wall structure and its size gradually increases due to blood flow in the artery. If the collecting system is affected, hematuria may occur and if the cyst breaks through the renal capsule, retroperitoneal hematoma may occur, forming a pulsatile hematoma. RAP rupture may be fatal in severe cases. The reported incidence rates of RAP following open partial nephrectomy and laparoscopic partial nephrectomy are 0.43 (4) and 0.97–1.7% (5,6), respectively. However, the incidence rate of RAP can be as high as 7.5% following laparoscopic partial nephrectomy for central renal tumors (7).

In the two present cases, RAP occurred several weeks following surgery. There are two possible reasons for this delay according to the current analysis (8). Firstly, the degradation of the absorbable suture may result in decreased tension at the ligation site in the kidney parenchyma and rehemorrhage of small blood vessels, such as the renal segmental arteries and arcuate artery, resulting in the formation of RAP. Secondly, blockage of the renal artery during surgery may make it difficult to identify the cross-sections of the small blood vessels. When the renal parenchyma is closed and the vascular clip on the renal artery is released, the tension of the suture does not completely block the blood flow into the small branches of the renal artery. Thus, blood clots may obstruct the small branches and result in rehemorrhage and formation of RAP long after patient discharge (6).

In open partial nephrectomy, the cross-sections of the blood vessels at the kidney wound can be identified and sutured more easily. However, in laparoscopy, the suturing of single small blood vessel sections is difficult and time-consuming, and the kidney is in a state of warm ischemia. Surgeons often close laparoscopic kidney wounds with continuous absorbable sutures to reduce renal warm ischemia time, and this may be one of the reasons why the incidence rate of RAP following laparoscopic partial nephrectomy is marginally higher than that following open surgery. With the widespread use of laparoscopic partial nephrectomy, the skillful surgical procedure and the secure suturing of small blood vessels at the kidney wound must be emphasized in order to reduce the incidence of RAP (9). A previous study also reported that the use of different hemostatic materials may aid in reducing the occurrence of RAP (10).

Although a patient with RAP may ultimately require total nephrectomy (11), selective embolization of the renal artery branch is currently the most effective treatment. Its achievement ratio is high (>80%) and has only a small influence on renal function, therefore, it remains the gold standard for the treatment of RAP (12).

In conclusion, the incidence rate of RAP following laparoscopic partial nephrectomy is low, but patients undergoing this surgery must be aware of this complication, particularly those with central renal tumors. Late onset of gross hematuria and lumbago on the affected side are the main symptoms of RAP. According to the results of the current study, the selective embolization of the renal artery branch is an effective treatment for RAP.

## References

[1] Ramani AP, Desai MM, Steinberg AP (2005). Complications of laparoscopic partial nephrectomy in 200 cases. J Urol.

[2] Winfield HN, Donavan JF, Godet AS, Clayman RV (1993). Laparoscopic partial nephrectomy: initial case report for benign disease. J Endourol.

[3] Ghoneim TP, Thornton RH, Solomon SB, Adamy A, Favaretto RL, Russo P (2011). Selective arterial embolization for pseudoaneurysms and arteriovenous fistula of renal artery branches following partial nephrectomy. J Urol.

[4] Albani JM, Novick AC (2003). Renal artery pseudoaneurysm after partial nephrectomy: three case reports and a literature review. Urology.

[5] Singh D, Gill IS (2005). Renal artery pseudoaneurysm following laparoscopic partial nephrectomy. J Urol.

[6] Zorn KC, Starks CL, Gofrit ON, Orvieto MA, Shalhav AL (2007). Embolization of renal-artery pseudoaneurysm after laparoscopic partial nephrectomy for angiomyolipoma: case report and literature review. J Endourol.

[7] Nadu A, Kleinmann N, Laufer M, Dotan Z, Winkler H, Ramon J (2009). Laparoscopic partial nephrectomy for central tumors: analysis of perioperative outcomes and complications. J Urol.

[8] Uberoi J, Badwan KH, Wang DS (2007). Renal-artery pseudoaneurysm after laparoscopic partial nephrectomy. J Endourol.

[9] Montag S, Rais-Bahrami S, Seideman CA, Rastinehad AR, Vira MA, Kavoussi LR, Richstone L (2011). Delayed haemorrhage after laparoscopic partial nephrectomy: frequency and angiographic findings. BJU Int.

[10] Pruthi RS, Chun J, Richman M (2004). The use of a fibrin tissue sealant during laparoscopic partial nephrectomy. BJU Int.

[11] Shakhssalim N, Nouralizadeh A, Soltani MH (2010). Renal artery pseudoaneurysm following a laparoscopic partial nephrectomy: hemorrhage after a successful embolization. Urol J.

[12] Inci K, Cil B, Yazici S (2010). Renal artery pseudoaneurysm: complication of minimally invasive kidney surgery. J Endourol.

